# Prodigiosin-Functionalized Probiotic Ghosts as a Bioinspired Combination Against Colorectal Cancer Cells

**DOI:** 10.1007/s12602-022-09980-y

**Published:** 2022-08-28

**Authors:** Nessrin Saleh, Hoda E. Mahmoud, Hoda Eltaher, Maged Helmy, Labiba El-Khordagui, Ahmed A. Hussein

**Affiliations:** 1 Department of Biotechnology, Institute of Graduate Studies and Research, Alexandria University, Alexandria, Egypt; 2 Department of Pharmaceutics, Faculty of Pharmacy, Alexandria University, Alexandria, 21521 Egypt; 3 Regenerative Medicine and Cellular Therapies Division, Faculty of Science, University of Nottingham, University Park, Nottingham, NG7 2RD UK; 4 Department of Pharmacology and Toxicology, Faculty of Pharmacy, Damanhour University, Damanhour, Egypt

**Keywords:** Probiotic ghosts, Bio-encapsulation, Prodigiosin, Colorectal cancer, Cell culture, HCT116 cells

## Abstract

*Lactobacillus acidophilus* ghosts (LAGs) with the unique safety of a probiotic, inherent tropism for colon cells, and multiple bioactivities offer promise as drug carriers for colon targeting. Our objective was to evaluate LAGs functionalized with prodigiosin (PG), apoptotic secondary bacterial metabolite, as a bioinspired formulation against colorectal cancer (CRC). LAGs were prepared by a chemical method and highly purified by density gradient centrifugation. LAGs were characterized by microscopic and staining techniques as relatively small-sized uniform vesicles (≈1.6 µm), nearly devoid of cytoplasmic and genetic materials and having a negatively charged intact envelope. PG was highly bound to LAGs envelope, generating a physiologically stable bioactive entity (PG-LAGs), as verified by multiple microscopic techniques and lack of PG release under physiological conditions. PG-LAGs were active against HCT116 CRC cells at both the cellular and molecular levels. Cell viability data highlighted the cytotoxicity of PG and LAGs and LAGs-induced enhancement of PG selectivity for HCT116 cells, anticipating dose reduction for PG and LAGs. Molecularly, expression of the apoptotic caspase 3 and P53 biomarkers in HCT116 intracellular proteins was significantly upregulated while that of the anti-apoptotic Bcl-2 (B-cell lymphoma 2) was downregulated by PG-LAGs relative to PG and 5-fluorouracil. PG-LAGs provide a novel bacteria-based combination for anticancer biomedicine.

**Graphical abstract:**

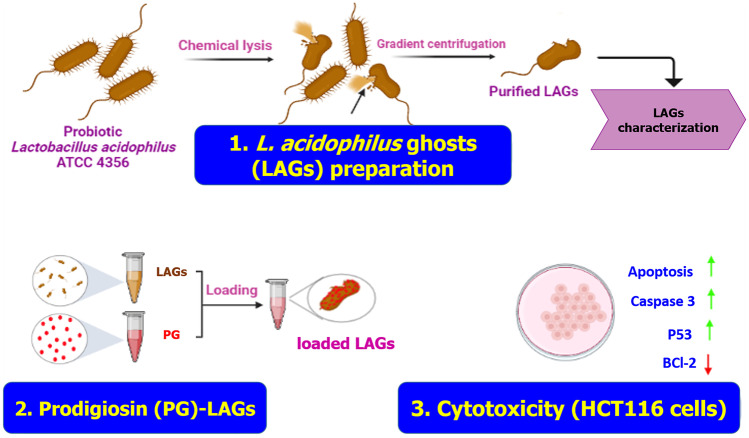

**Supplementary Information:**

The online version contains supplementary material available at 10.1007/s12602-022-09980-y.

## Introduction

Bacteria have attracted great research attention in biomedicine as both bioactive agents and drug carriers [1], taking advantage of their sustainability, rapid proliferation, easy genetic and technological manipulation, and biotargeting ability. Different strategies have been established recently to enhance the therapeutic efficacy of bacteria by increasing their bioactivity, survival in the biological milieu, safety, and targeting properties. Strategies documented to date include mainly surface functionalization [2, 3], protection and activation by diverse materials [4–6], and modification for controlling intra-tumoral drug distribution [7]. As drug carriers, bacteria proved to effectively deliver therapeutic agents for treating cancer and other conditions [7–9]. Apart from live bacteria, progress in biotechnology has put forward bacterial structures and derivatives as bioinspired drug carriers. Among these, bacteria-derived vesicles such as outer membrane vesicles (OMVs) [10], bacteriosomes [11], and bacterial ghosts [12] have been explored as non-living, non-denatured bacterial structures of benefit in biomedical applications.

Bacterial ghosts (BGs) offer an emerging biotechnological platform in the prevention and treatment of disease. BGs are intact envelopes of bacterial cells, generally emptied of their cytoplasmic and genetic materials by gentle poring methods. While the genetic method was commonly used for the preparation of Gram-negative bacterial ghosts [13, 14], chemical methods allowed the generation of BGs of both Gram-negative and Gram-positive bacteria [15, 16]. BGs retain the cellular morphology and surface structural components of native bacterial cells, principally the antigenic proteins for recognition by the immune system, as well as fimbriae and adhesins to facilitate targeting and binding to different cells and tissues [13, 17]. Such outstanding surface features endow BGs with the ability to stimulate immune responses and serve as vectors for the delivery of human and veterinary vaccines [18–20]. Equally important, BGs exert therapeutic potential on their own and may synergize the activity of other drugs via BG-induced immunostimulatory effects [21]. Compared with biomaterial-based drug carriers, PGs are safer and more stable in the biological milieu and are characterized by a large internal space and inherent drug biotargeting ability [11, 22]. Recently, promising effects have been obtained with BG-mediated delivery of anticancer drugs [23, 24] and macrophage delivery of anti-infective agents [25].

However, the bacteria used in the preparation of BGs to date are mostly pathogenic, and using non-pathogenic bacteria would greatly improve safety. Gram-positive lactic acid probiotic bacteria that have significant health-promoting effects are generally recognized as safe (GRAS) and have shown immunomodulatory capability [26]. Several anticancer effects have also been linked to their structural components and metabolites [27, 28]. Nevertheless, literature reports on the use of *Lactobacillus acidophilus* (LA) and other probiotic ghosts as drug carriers are scarce. One study reported the use of *Lactobacillus casei* ghosts as a potential carrier for DNA vaccines [29]. Among the best known lactic acid bacteria, LA naturally colonizes the human colon with resilient adhesive properties as a result of binding to mucin [30] and recognition of their antigens by colon cells and the adjacent immune system [31]. Such distinct properties render LA ghosts (LAGs) highly promising as drug carriers for targeting colorectal cancer (CRC) cells.

To date, drug delivery by BGs has been generally restricted to chemotherapeutic agents such as 5-fluorouracil [24] and doxorubicin [23]. Functionalizing BGs with bacterial metabolites having colorectal cancer inhibitory activity may establish a new microbially derived biotargeted platform for the treatment of CRC, one of the most common malignancies worldwide. In this regard, prodigiosin (PG), a secondary metabolite of *Serratia marcescens*, induces significant cytotoxicity in a variety of cancer cell lines [32] without inducing marked toxicity in nonmalignant cell lines [33]. PG’s selective anti-colorectal actions occur via apoptosis by altering the expression of apoptosis-related genes [34, 35] and restoration of p53 tumor suppressor activity in chemo-resistant CRC stem cells [36]. In CRC stem-like cells, PG reduces survivin levels while increasing caspase-3 and miRNA-16–1 levels [33, 34]. Besides, PG sensitizes CRC cells to cell death induced by anticancer drugs [33, 37].

The objective of this study was to produce highly purified LAGs and provide a proof of concept for their application as a novel bioinspired drug carrier against CRC. Native LAGs were prepared, purified, and characterized as new probiotic Gram-positive ghosts. LAGs were functionalized with PG to develop a novel bacteria-derived delivery system (PG-LAGs) combining the potentials of LAGs and PG. The activity of PG-LAGs against HCT116 CRC cells was assessed at the cellular and molecular levels relative to 5-fluorouracil, a standard chemotherapeutic agent.

## Materials and Methods

### The Development of *Lactobacillusacidophilus* Ghosts

#### Preparation

*L. acidophilus* (LA) ATCC 4356 was obtained from WFCC-MIRCEN-World Data Centre for Microorganisms (http://www.wdcm.org/), Faculty of Agriculture, Ain Shams University, Cairo, Egypt. De Man, Rogosa, and Sharpe (MRS) broth and agar were used for growing and preserving LA (HiMedia Laboratories, India). LAGs were prepared using essentially the chemical sponge-like reduced protocol (SLRP) [38] with some modification. In brief, 100 mL of MRS medium in a 250-mL Erlenmeyer flask was inoculated with one mL of overnight LA culture and incubated at 37 °C for 72 h until the late stationary phase [39] with an OD_600_ = 8 [40]. Bacterial cells were harvested by centrifugation at 672 × *g* for 10 min, and pellets were washed twice with 0.5% NaCl. For the chemical treatment experiment, washed LA cells were adjusted to OD600 = 0.9 and incubated in a mixture of NaOH at the minimum growth concentration (MGC), 0.01% and sodium dodecyl sulfate at the minimum inhibitory concentration (MIC), and 0.1% with shaking at 120 rpm overnight (16–18 h) at 37 °C. Bacterial pellets were harvested by centrifugation at 672 × *g* for 10 min and washed twice with 0.5% NaCl solution. Chemical treatment was applied to bacterial cells harvested at different stages of growth (exponential phase, stationary phase, and late stationary phase).

Differentiation of LAGs from un-evacuated LA cells was achieved using toluidine blue staining and transmission electron microscopy (TEM, JEOL-JSM-1400 PLUS). For staining, a bacterial suspension and a ghost suspension were smeared on the surface of a glass slide and fixed by gentle heating. The slides were stained with toluidine blue dye (0.1%) for 15 min, washed with a few drops of water, air-dried, and examined under the oil immersion lens of a light microscope (Olympus CX31 Microscope). Live LA cells and LAGs were examined by TEM as reported [41].

#### Purification

The LAGs were purified by separation from live and dead un-evacuated LA cells by subjecting the resuspended bacterial mass to a density gradient centrifugation technique [42] for different time intervals (6, 9, 12, 15, and 18 min) at 168 × *g*. This was followed by centrifugation of each separated fraction for 20 min at 672 × *g* to precipitate LAGs. To assess the efficiency of the separation procedure, the purified LAGs obtained at the end of the centrifugation process were examined by light microscopy following toluidine blue staining and TEM imaging. For quantification of the recovered LAGs in the fractions obtained by gradient centrifugation at the different time intervals, samples were stained with toluidine blue to visualize live LA cells under the oil immersion lens of the light microscope. More quantitative data were obtained by analyzing captured images using ImageJ software (ImageJ v1.53 k).

#### Characterization

LAGs were examined in comparison with live LA cells for morphology and elimination of cytoplasmic and genetic materials in addition to the integrity and negativity of the cell envelope. The morphology of LAGs in comparison with live LA cells was examined by scanning electron microscopy (SEM, JSM-5300 (JEOL). The expulsion of intracellular content was checked by TEM [41], and elimination of genetic materials and total protein was assessed by gel electrophoresis.

DNA was extracted using G-spin™ Total DNA Extraction Kit (iNTRON, Co. Korea). The isolated DNA was subjected to 1% agarose gel electrophoresis and visualized under a UV-transilluminator. The concentration of isolated DNA from live LA and LAGs was measured spectrophotometrically using the NanoDrop 2000 Spectrophotometer-Thermo (Fisher Scientific™) [43].

The protein content of the cytoplasm and the envelope (cell wall and cell membrane) including the S-layer surface structural component [44, 45] was estimated using the Bradford method [46] and BSA as a standard protein. All protein samples were subjected to 12% SDS-PAGE as reported [47]. The value of each protein band was estimated using the TotalLab Analysis Software (ver.1.0.1) and the UVP GelDoc-Ite gel documentation system. The integrity of the LAG capsule was assessed by light microscopy following staining with crystal violet/copper sulfate (Anthony’s stain) consisting of crystal violet as the primary stain and 20% copper sulfate solution as a decolorizing solution and counter stain [48]. Finally, the LAG surface negativity was examined using nigrosine staining, performed by mixing a small drop of nigrosine solution (10% w/v) with a small drop of bacterial or ghost suspension near the end of a glass slide. The mixture was spread into a thin smear and examined under the oil immersion lens of a light microscope after air drying [49].

### The Development of Prodigiosin-Functionalized *L. acidophilus* Ghosts

#### Preparation

Prodigiosin, prepared and characterized earlier [50], was used. Ghost pellets were incubated with 1 mL PG solution (6 mg/mL) in a methanol:acetic acid (1:1) solvent system with gentle shaking at ambient temperature (≈ 28 °C) for 2 h. The PG-LAGs were separated by centrifugation at 24192 × *g* for 5 min, washed twice using 0.5% NaCl, and stored at 4 °C suspended in 0.5% NaCl. The effect of the solvent system composition (methanol:acetic acid ratios 1:0, 1:1, and 3:1), PG concentration (0.1–6 mg/mL), and incubation time (0.5–5 h) at 200 rpm agitation rate on PG loading was investigated. For drug payload determination, PG was extracted from LAGs by vigorous shaking with methanol for 10 min and assayed by UV–Vis spectrophotometry at *λ*_max_ 535 nm. The % loading efficiency (LE) was calculated as follows:

$$\mathrm{Loading\, efficiency}= \frac{\mathrm{Entrapped\, PG } }{\mathrm{}\mathrm{LAGs\, weight }}\times100$$

To prove the entrapment stability of PG within LAGs, PG-LAGs were washed eight times with 0.5% NaCl, separated by centrifugation at 24192 × *g* for 5 min, and the supernatant analyzed for PG spectrophotometrically at *λ*_max_ 535 nm.

Entrapment of PG in LAGs was verified by comparing PG-LAGs with native LAGs by digital photography, light microscopy without staining, TEM, and confocal fluorescence microscopy (CFM) using a Leica TCS SPE Confocal Microscope with a Leica LAS X interface at the excitation and emission wavelengths of 543 nm and 570 nm, respectively.

### Physical Characterization of PG-LAGs

The size, polydispersity index (PDI), and zeta potential (ZP) of PG-LAGs in comparison with native LAGs were measured by dynamic light scattering (DLS) using Zetasizer Nano ZS (Malvern Zeta Sizer, UK).

#### PG Release Studies

The in vitro release of PG from PG-LAGs was studied at 37 °C by a dialysis method [51] using acetate buffer pH 5.5 and phosphate buffer saline (PBS) pH 7.4 with or without the addition of 5% methanol or up to 3% Tween 80 as release media. Briefly, 2.5 mL of PG-LAGs suspension in 0.5% saline was placed in a dialysis bag (VISKING^®^ dialysis tubing MWCO 12,000–14,000) and shaken in 30 mL of the release medium at 100 rpm for 48 h protected from light. In addition, PG release was examined in simulated gastrointestinal fluids by immersing PG-LAGs in simulated gastric fluid (SGF, 10 mL pepsin/HCl, 320 mg/100 mL, pH 1.2) for 2 h, followed by immersion in simulated intestinal fluid (SIF, 10 mL of pancreatin/PBS, 1 g/100 mL, pH 7.2) for 4 h. Samples of the release medium (2 mL) were withdrawn for analysis at different time intervals and replaced with 2 mL of fresh medium at 37 °C. The concentration of PG released was determined by UV–Vis spectrophotometry at *λ*_max_ 535 nm. In addition, PG-LAGs were visualized at the beginning and the end of the 48 h release experiment in PBS pH 7.4 containing 3% Tween 80 as the release medium by CFM.

#### Cytotoxicity Studies

The cytotoxicity of PG-LAGs in comparison with native LAGs, PG, and 5-fluorouracil (5-FU) was assessed using HCT116 colorectal cancer (CRC) cell line and normal human fibroblasts (NHFs, American Type Culture Collection (ATCC, USA). The cells were cultured in DMEM (Dulbecco’s Modified Eagle Medium) supplemented with 10% FBS (fetal bovine serum) and 1% penicillin/streptomycin and maintained in an incubator with 5% carbon dioxide and humidified air at 37 °C. Stock solutions of PG and 5-FU in DMSO (dimethylsulfoxide) and stock suspensions of live LA cells, native LAGs, and PG-LAGs were diluted in DMEM to reach the required concentrations. DMEM containing the same amount of DMSO used in treatment groups (1%) was used as control. Cell viability was assessed by the MTT (3-(4,5-dimethylthiazol-2-yl)-2,5-diphenyl-2H-tetrazolium bromide) assay [52] following a 24 h incubation period of cells seeded in 96-well plates (4000 cell/well) with the test preparations. These included PG solution (0.25–10 μg/mL), 5-FU solution (0.25–10 μg/mL), suspensions of LAGs (37.5–1500 μg/mL), and PG-LAGs (1:25 with PG concentration of 0.25–10 μg/mL). The medium was discarded and the cells were incubated with 20 μL MTT reagent (5 mg/mL) for 4 h. Formazan crystals were dissolved in 150 μL of DMSO and the absorbance at 590 nm was recorded using a Bio-Rad microplate reader.

#### Cell Viability, IC50 Values, and Selectivity Index

The % viability of HTT116 cells was determined in triplicate relative to the control wells. The median inhibitory concentrations (IC50) were determined using CompuSyn software (CompuSyn, Inc., version 1) according to the Chou-Talalay method [53]. The selectivity of PG-LAGs for HCT116 CRC cells in comparison with LAGs, PG, and 5-FU was assessed using the selectivity index (SI) calculated as follows [52]:

$$\mathrm{SI}= \frac{\mathrm{IC}50\,\mathrm{ in\, normal\, fibroblasts}}{\mathrm{IC}50\,\mathrm{ in\, HCT}\,116\,\mathrm{ CRC\, cell\, line}}$$

For the analysis of the combinatorial effects of PG and LAGs in the PG-LAGs combination on the HCT116 CRC cells, the type of interactive effect of PG and LAGs was examined by determining the combination index (CI) and dose reduction index (DRI) using CompuSyn software [53].

## Analysis of HCT116 Cells Apoptosis Following the Incubation with LAGs

Cell lysates were obtained using RIPA (radioimmunoprecipitation assay) lysis and extraction buffer (Thermo Scientific, USA (#89,900) containing 25 mM Tris–HCl pH 7.6, 150 mM NaCl, 1% NP-40 (nonyl phenoxypolyethoxylethanol), 1% sodium deoxycholate, and 0.1% SDS. HCT116 cell pellets were mixed with 1 mL RIPA buffer (containing a protease inhibitor cocktail), shaken gently for 15 min on ice, and centrifuged at 14000 × *g* for 15 min to pellet the cell debris. The supernatants were separated and stored at −20 °C pending determination of the total extracted cellular protein using the Bradford assay [46].

The level of active caspase-3 in cell lysates was determined using a colorimetric kit (# ab39401, Abcam) [52]. A p-nitroaniline moiety released after hydrolysis of the peptide substrate (Ac-DEVD-pNA) by active caspase-3 in cell lysates was quantified using a calibration curve constructed from absorbance at 405 nm measured on a microtitre plate. Data are the mean ± SEM of three replicas. The levels of P53 and Bcl-2 proteins per gram of total cellular protein in the cell lysate were determined using Human Immunoassay Elisa kits (ab171571-p53 Human SimpleStep ELISA^®^ Kit) and (ab119506 – Bcl-2 Human ELISA^®^ Kit). The p53 and Bcl-2 protein levels were normalized by cell viability for each treatment. Three replicas were performed for each protein and the mean ± SEM was calculated.

### Statistical Analysis

Data were analyzed using Graph Pad Prism^®^ version 6 software (GraphPad Software Inc., CA, USA). Multiple comparisons were analyzed by one-way analysis of variance (ANOVA) and post hoc Tukey’s multiple comparison test. Data expressed as mean ± SD is representative of three measurements. A value of *p* < 0.05 indicated significance.

## Results

### The Development of *L. acidophilus* Ghosts

#### Preparation

**Fig. 1:**
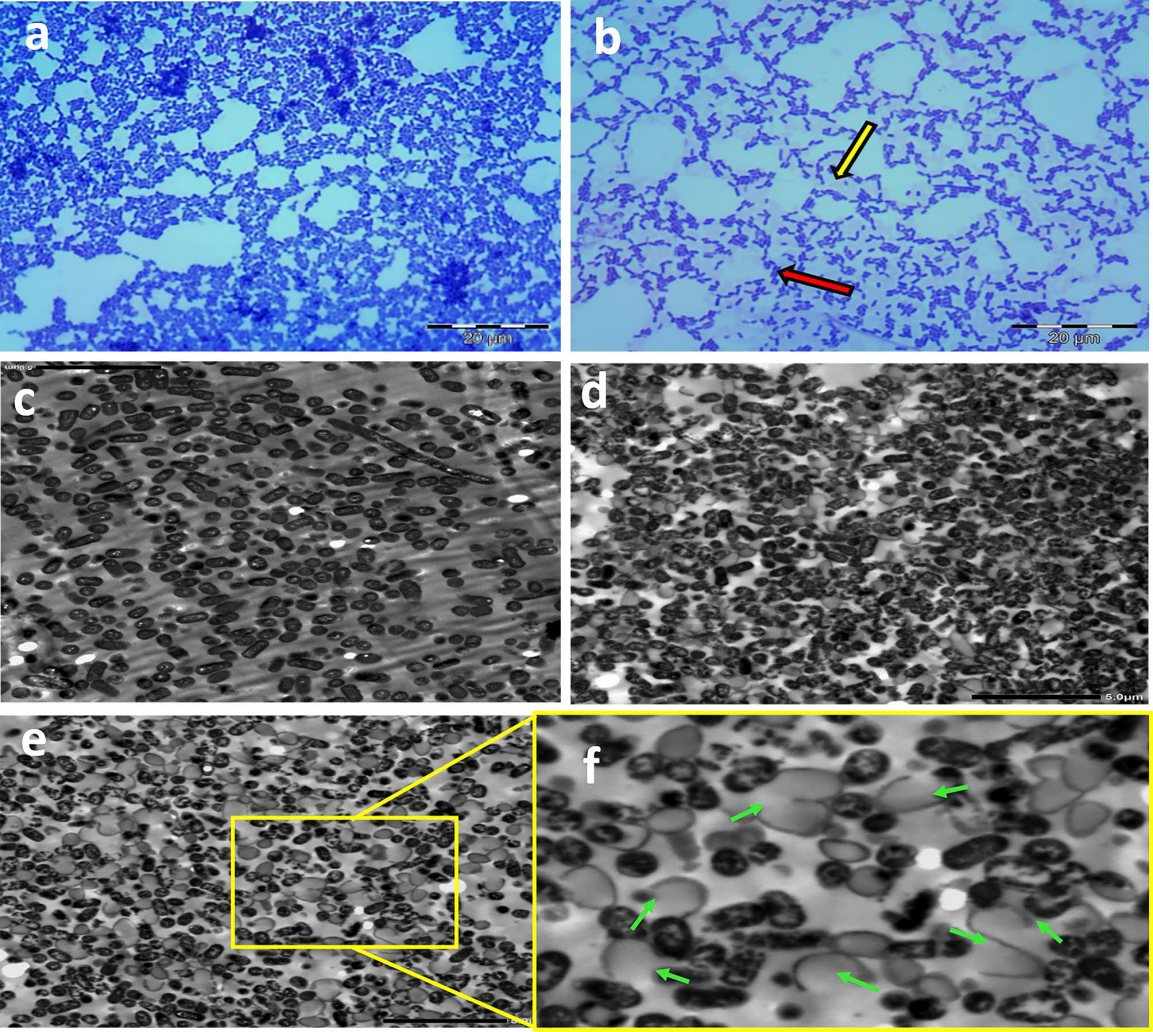
Microscopic examination of Live *L. acidophilus* (LA) cells and the bacterial mass consisting of LA cells (live and dead un-evacuated) and *L. acidophilus* ghosts (LAGs). **a**, **b** Light microscopic images at 100 × following toluidine blue staining of (**a**) live LA cells and **b** bacterial mass. The red and yellow arrows point to groups of stained un-evacuated LA cells and unstained LAGs, respectively; **c**–**f** TEM of (**c**) live LA cells, **d**, **e** bacterial mass obtained after chemical treatment under shaking and static conditions, respectively (1500 ×); **f** Enlarged section of **e** showing deteriorated LAG cell envelope (green arrows)

The chemical sponge-like reduced protocol (SLRP) using NaOH and SDS generated a bacterial mass consisting of bacterial LAGs, dead un-evacuated LA cells, and the remaining live LA cells which tolerated chemical lysis. The harvested pellets obtained in the exponential and stationary phases (after 24 and 48 h) were sticky and LAGs showed complete breakdown of the cell envelope. However, the bacterial mass harvested in the late stationary phase contained 33% of LAGs approximately. LAGs were differentiated from LA cells in the mass using a new light microscopy method based on toluidine blue, known to bind to DNA. Figure 1 indicated that live LA cells acquired the intense toluidine blue color (Fig. 1a) while the presence of LAGs, presumably devoid of genetic materials, led to an obvious reduction in the blue staining of the bacterial mass (Fig. 1b). Comparison of intracellular content of live LA cells (Fig. 1c) and the bacterial mass (Fig. 1d) by TEM verified the presence of a mixture of evacuated LAGs and un-evacuated LA cells in the mass. Attempts to increase the yield of LAGs by increasing the concentration of NaOH and SDS or applying the chemical treatment under static conditions resulted in rupture of the ghost cell envelope (Fig. 1e, f).

#### Purification

**Fig. 2:**
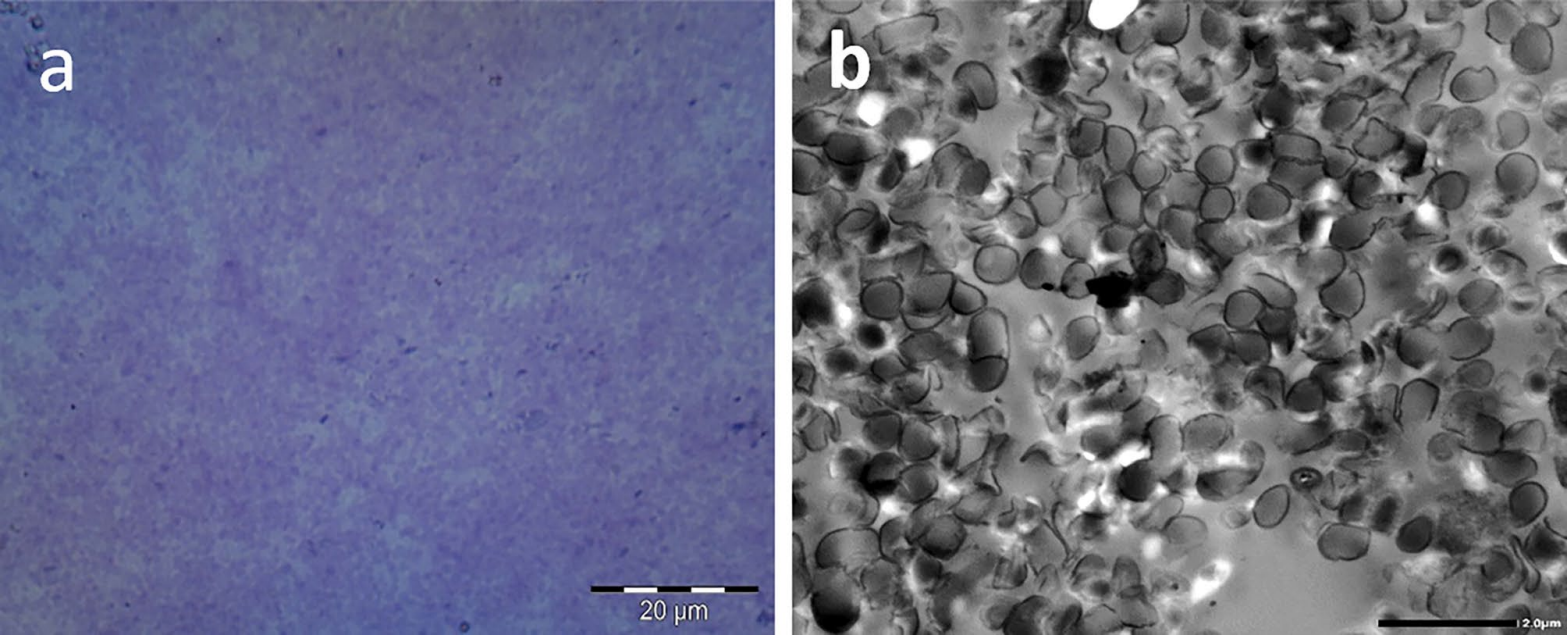
Purified *L. acidophilus* ghosts. **a** Light microscopic image following toluidine staining at 100 × and **b** TEM at 3000 ×

Subjecting a suspension of the bacterial mass in 0.5% NaCl to a density gradient centrifugation process involving six cycles of repeated centrifugation resulted in effective separation of light-weight LAGs. The first five cycles at 168 × *g* for 6, 9, 12, 15, and 18 min were effective in eliminating the heavy dead un-evacuated LA cells and the remaining live cells. The sixth cycle for 20 min at 672 × *g* allowed precipitation of the lighter bacterial ghosts. As shown in Fig. 2, light microscopy (Fig. 2a) and TEM (Fig. 2b) imaging verified the absence of LA cells. In addition, quantitative ImageJ counting of toluidine blue-stained LA cells revealed that the first to fifth centrifugation fractions contained 36.0%, 69.2%, 75.0%, 88.3%, and 91.3% of LAGs, respectively. The increase in LAGs content after the sixth centrifugation cycle to 99.9% indicated high purity.

### Characterization

#### Morphology

**Fig. 3:**
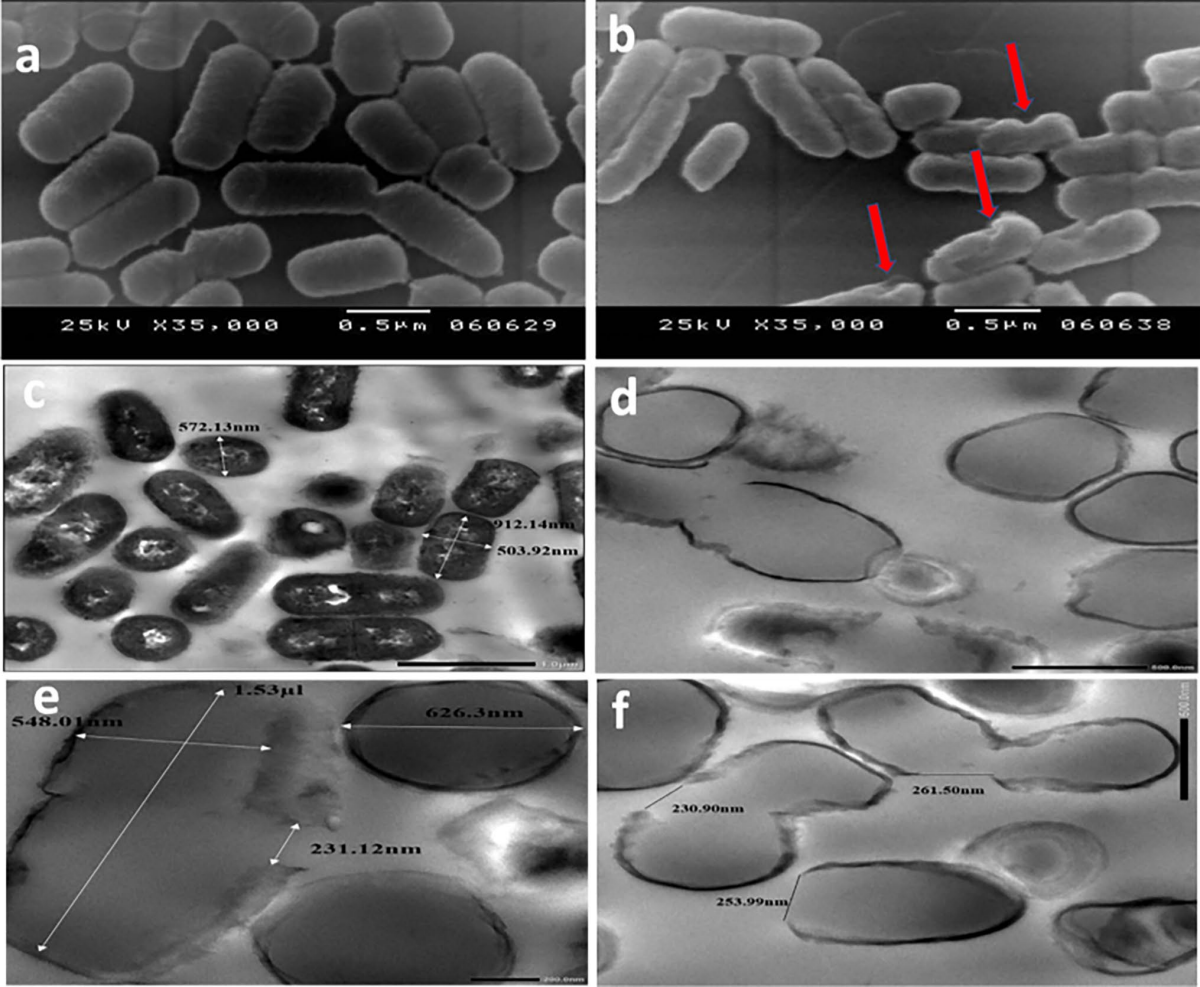
Electron microscopic characterization of *L. acidophilus* ghosts (LAGs) in comparison with live LA cells. Scanning electron micrographs at 35000 × for **a** live *L. acidophilus* cells and **b** LAGs. Red arrows point to the pores from which cytoplasm and genetic materials were expelled. Transmission electron micrographs for **c** live LA cells at 8000 × and **d** LAGs at 15000 × showing expulsion of cytoplasmic content; **e** LAGs at 20000 × showing internal dimensions and **f**: LAGs at 15000 × showing pore size

SEM at 35000 × of live LA cells (Fig. 3a) and purified LAGs (Fig. 3b) showed that LAGs were intact and similar to live cells except for the existence of pores from which the intracellular contents were expelled. Compared with live LA cells (Fig. 3c), LAGs appeared empty with retention of a cohesive cell envelope (Fig. 3d–f) as indicated by TEM. The calculated internal volume of LAGs was 0.13 µm^3^ approximately and pores with a mean size of 153.63 ± 12.23 nm were observed at the division sites representing the weak point of the bacterial wall (Fig. 3e, f).

#### Residual Genetic Materials and Proteins

The extracted DNA from live LA and LAGs was electrophoresed on a 1% agarose gel. As shown by supplementary Fig. 1a, no band was visible in lane 2 specific to DNA isolated from LAGs when compared to lane 1 specific to DNA isolated from live LA. As determined by Nanodrop^®^ spectrophotometry, the concentration of released intracellular DNA was 111.6 ng/µL and 13.8 ng/µL for live LA and LAGs, respectively, implying loss of most of the intracellular DNA.

Expulsion of the total protein content of live LA and proteins of LA envelopes was verified by SDS-PAGE electrophoresis (supplementary Fig. 1b). The values for each protein band are tabulated in the supplementary Table 1. The total protein profile of live LA (Lane 1) compared to that of LAGs (lane 2), indicated loss of cytoplasmic materials from LAGs. In addition, lane 3 for the protein profile of the supernatant after chemical treatment revealed expulsion of the LAGs proteins into the supernatant. SDS-PAGE bands at 46 KDa and 49 KDa indicated preservation of the S-layer protein in LAGs similar to live LA cells.

#### Integrity and Negativity of the *L. acidophilus* Ghost Envelope

**Fig. 4:**
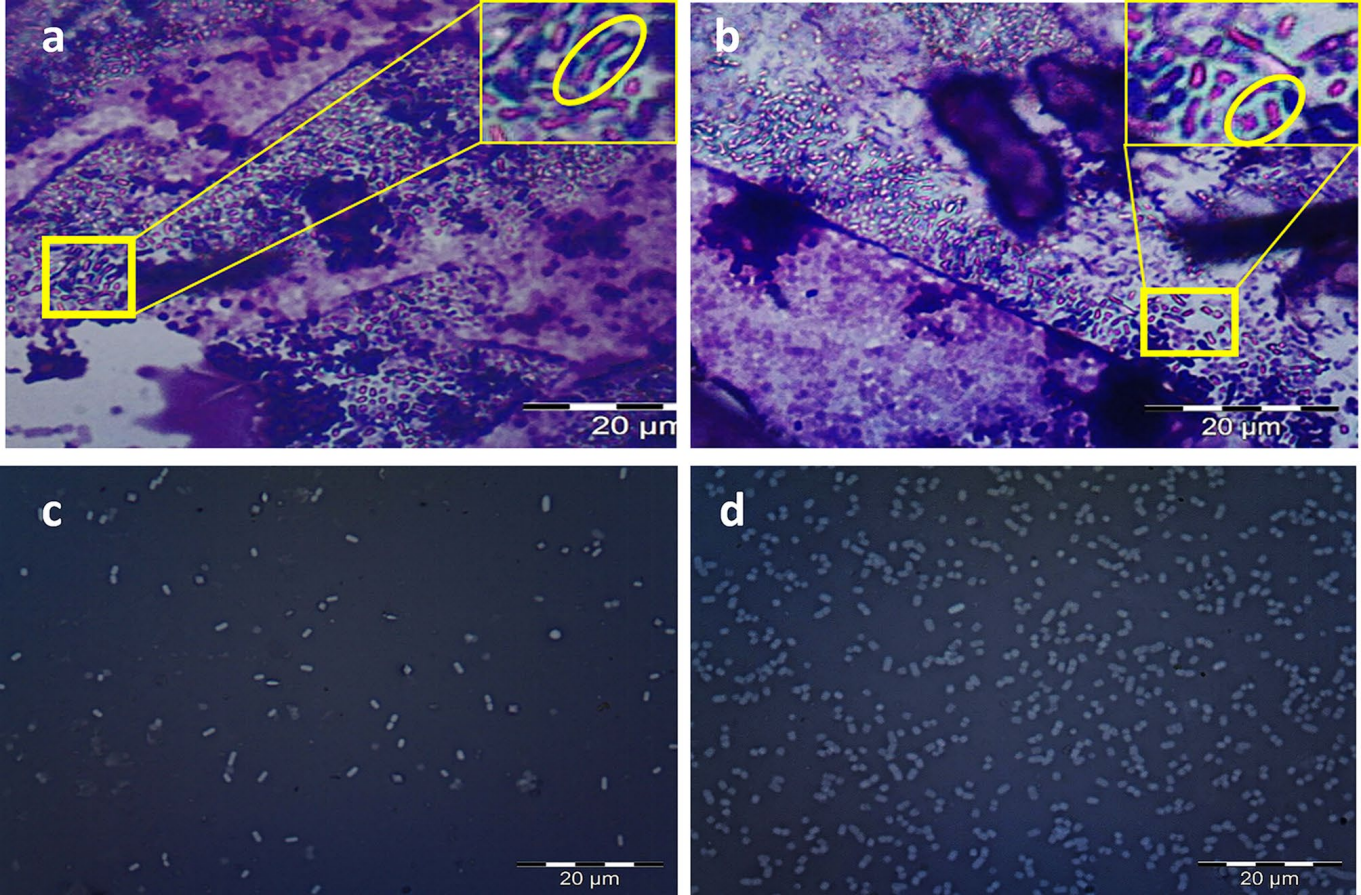
Light microscopic examination of the integrity and surface negativity of *L. acidophilus* ghosts (LAGs) in comparison with live LA cells at 100 × magnification. Crystal violet/copper sulfate staining of (**a**) live LA cells and **b**: LAGs. The insets in **a** and **b** show enlarged sections (yellow oval shape) highlighting live LA cells and LAGs respectively with intact capsule appearing as faint blue halos around the purple cell. Nigrosine staining revealed (**c**) live LA cells and **d** LAGs as bright halos on the dark background

The integrity of LAGs envelope was demonstrated by light microscopy imaging of LAGs in comparison with live LA cells following crystal violet/copper sulphate (Antony’s stain) staining of the capsule (blue) and crystal violet staining of the cells (purple) (Fig. 4). The Antony’s staining revealed the existence of an intact capsule (blue halo) in both live bacteria (Fig. 4a) and LAGs (Fig. 4b). This was verified by the enlarged insets in Fig. 4a, b showing a blue capsule surrounding purple live bacteria cells and LAGs. Preservation of the negativity of live LA cell wall (Fig. 4c) and LAGs (Fig. 4d) after chemical treatment was affirmed by nigrosine background staining [49]. The negatively charged nigrosine stain did not penetrate the bacterial cell wall and obliterated the background leaving the organisms as bright and visible halos in the darkened field.

### The Development of Prodigiosin-Functionalized *L. acidophilus* Ghosts

#### Preparation

Loading of PG by incubating native LAGs with PG solution at 200 rpm in a methanol: acetic acid solvent system was affected by the PG concentration, solvent system composition, and incubation time. Preliminary experiments indicated that a methanol: acetic acid (1:1) blend and 2 h incubation at 200 rpm allowed for maximum PG loading. Increasing PG concentration solution under these conditions increased PG payload which was determined by vigorous shaking of PG-LAGs with methanol for 5 min. Supplementary Fig. 2 shows the profile of increase in PG loading as a function of the initial PG concentration. To verify entrapment stability of PG within LAGs, PG-LAGs were washed eight times with 0.5% NaCl and centrifuged at 24192 × *g* for 5 min. The PG concentration in all supernatants did not reach a detectable level when assayed spectrophotometrically at *λ*_max_ 535 nm.

#### Physical Characterization

The size of native LAGs determined by dynamic light scattering was 1.13 ± 0.13 µm with a PDI 0.15 ± 0.09 while that of PG-LAGs was 1.59 ± 0.24 µm with PDI 0.27 ± 0.04. ZP measurements revealed a relatively low negative surface charge for native LAGs (− 4.20 ± 4.22 mV) and PG-LAGs (− 0.821 ± 4.03 mV).

#### Verification of PG Entrapment in LAGs

**Fig. 5:**
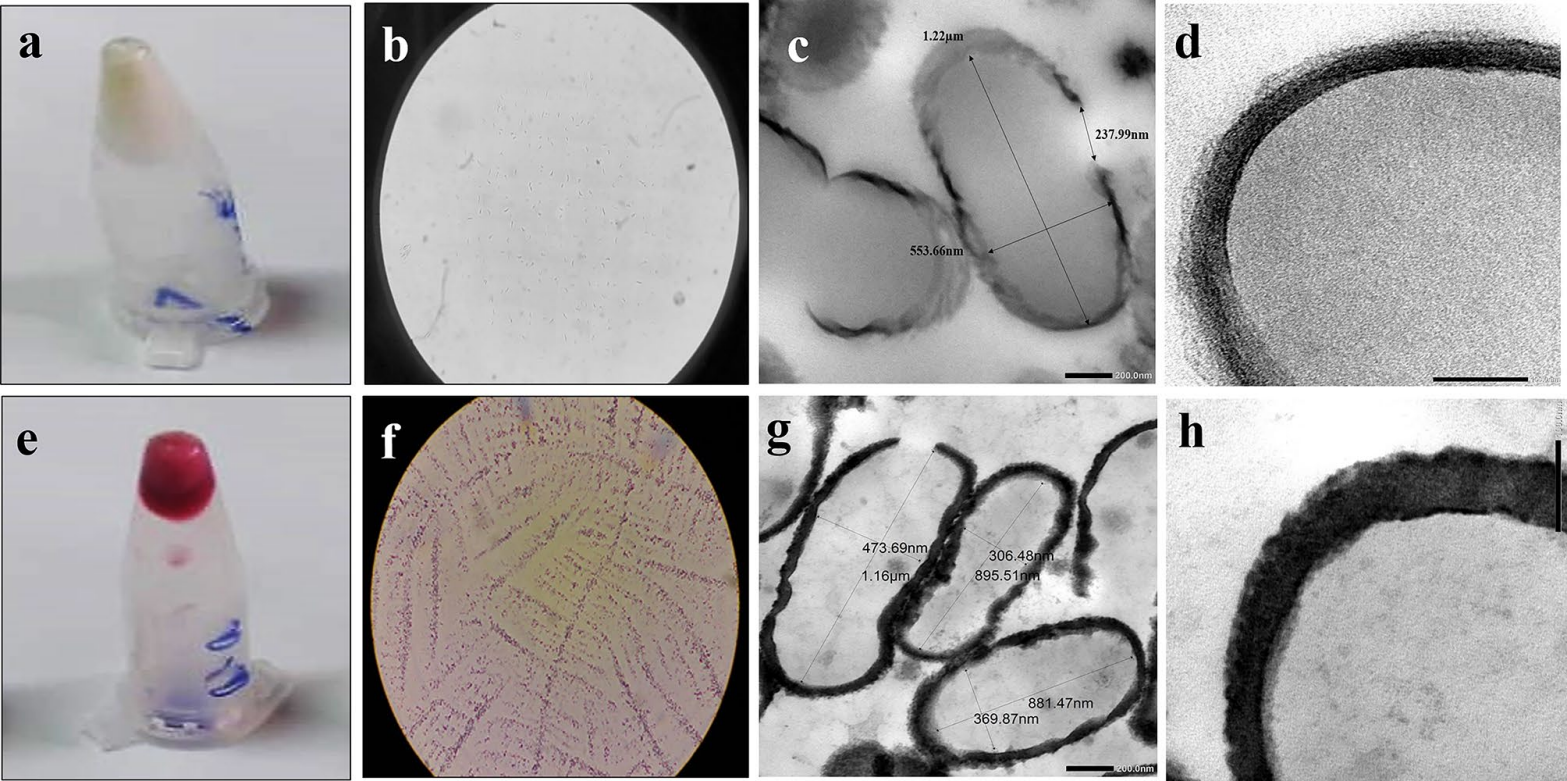
Images of native *L. acidophilus* ghosts (LAGs, **a**–**d**) and prodigiosin-loaded LAGs (PG-LAGs, **e**–**h**). Digital photos of LAG and PG-LAG pellets in inverted Eppendorf tubes (**a**, **e** respectively). Images of LAGs and PG-LAGs under the oil immersion lens of the light microscope without staining (**b**,** f** respectively). Transmission electron micrographs for LAGs and PG-LAGs at 20,000 × (**c**, **g** respectively) and at 80,000 × (**d**, **h** respectively)

**Fig. 6:**
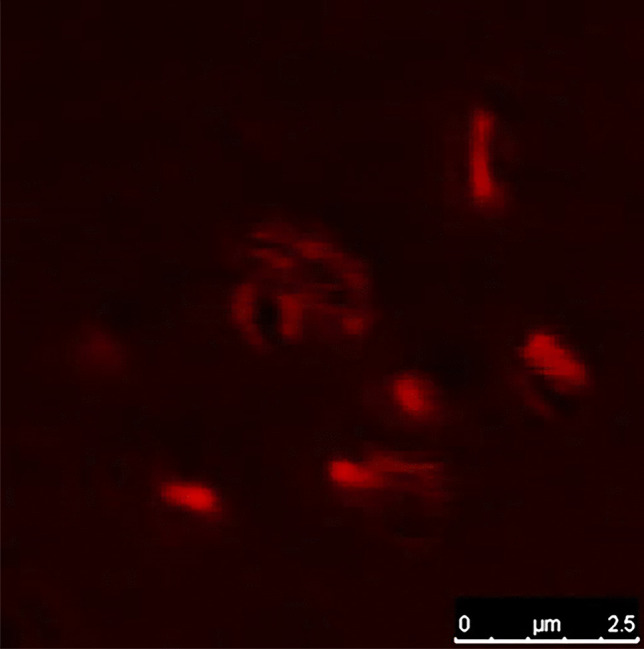
Confocal fluorescence microscopy image of prodigiosin-loaded bacterial ghost cells

PG entrapment in LAGs was verified by digital photography, light microscopy, and TEM (Fig. 5a–h). The white pellet of native LAGs (Fig. 5a) acquired the characteristic PG red color upon loading (Fig. 5e). PG-LAGs also appeared as red vesicles under the 100 × lens of the light microscope without staining (Fig. 5b, f). TEM imaging at 20,000 × revealed that the density of the cell envelopes of native LAGs (Fig. 5c) was obviously increased in PG-LAGs (Fig. 5g), with maintenance of the calculated internal volume (≈ 0.13 µm^3^) unchanged. TEM also demonstrated binding of PG to the LAG cell envelope as affirmed by images of LAGs and PG-LAGs at 80,000 × (Fig. 5d, h). In addition, CFM imaging the PG-LAGs proved the presence of PG in the cell envelope (Fig. 6). Quatitatively, the loading efficiency was 4%.

#### Prodigiosin Release Studies

**Fig. 7:**
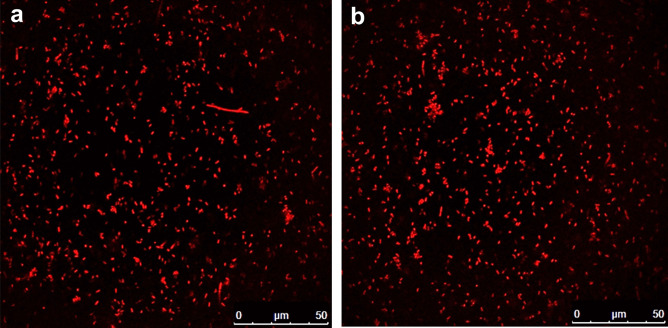
Confocal fluorescence microscopy images of prodigiosin-loaded bacterial ghost cells at the beginning (**a**) and the end (**b**) of a 48 h release experiment in PBS pH 7.4 containing 3% Tween 80 at 37 °C

Data for PG release from PG-LAGs at 37 °C in media of different pH (acetate buffer pH 5.5 and phosphate buffer saline pH 7.4 with or without the addition of 5% methanol at 100 rpm for 48 h indicated PG retention by LAGs. Inclusion of up to 3% Tween 80 in PBS pH 7.4 did not enhance PG release. Lack of PG release was also observed when simulated gastric and simulated intestinal fluids were used as release media. CFM imaging of PG-LAGs at the beginning and the end of the 48 h release experiment using PBS pH 7.4 containing 3% Tween 80 (Fig. 7) affirmed retention of PG fluorescence by LAGs. Quantitatively, retention of PG (93.15 ± 8.8%, *n* = 3) recovered at the end of the experiment provided evidence for PG binding to LAG.

### Cytotoxicity Studies

#### Cell Viability, IC50, and Selectivity Index

**Fig. 8:**
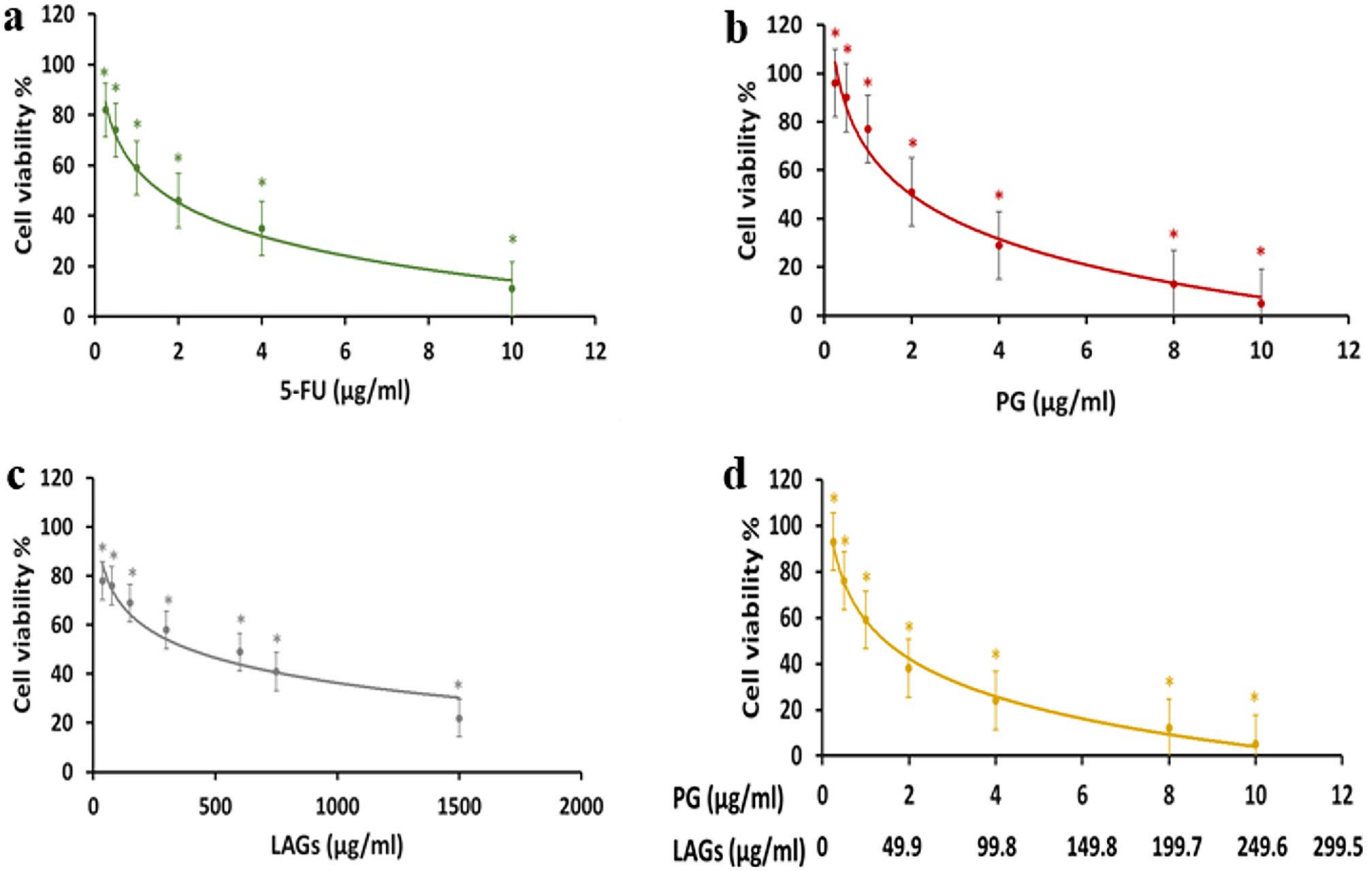
The effect the test preparations on the viability of HCT116 cells. The % viability of HCT116 cells determined using the MTT assay upon treatment with (**a**) 5-fluorouracil (5-FU), **b** prodigiosin (PG), **c** *L. acidophilus* ghost (LAGs), and **d** PG-loaded ghosts (PG-LAGs) at increasing concentrations for 24 h. Data points represent the mean ± SEM (*n* = 3). *p < 0.05 indicates a significant difference *vs* the corresponding control group

Cytotoxicity data expressed as % viability of HCT116 CRC cells upon exposure to the test preparations at increasing concentrations for 24 h at 37 °C using MTT assay are shown in Fig. 8a–d. Cell viability curves for 5-fluorouracil (5-FU) (Fig. 8a), PG (Fig. 8b), LAGs (Fig. 8c), and PG-LAGs (Fig. 8d) showed a concentration-dependent effect within their respective concentration ranges.

The IC50 values computed from cell viability data (Table 1) indicated that the IC50 of PG was slightly higher than that of 5-FU with a non-statistically significant difference (*p* > 0.05). Combining PG and LAGs in PG-LAGs induced an 8.5-fold reduction in the IC50 of LAGs. The selectivity of the test preparations for HCT116 CRC cells expressed as the selectivity index (SI) (IC50 values in normal human fibroblasts relative to those in HCT 116 cells) revealed significantly greater (*p* < 0.05) selectivity of PG (SI 17.87), LAGs (SI 28.24), and PG-LAGs (SI 43.60) for HCT116 cells relative to 5-FU (SI 8.63). Furthermore, the SI index of PG and LAGs was significantly increased (*p* < 0.05) by their combination in PG-LAGs.

**Table 1:** IC50 values for 5-fluorouracil (5-FU), prodigiosin (PG), *L. acidophilus* ghosts (LAGs), and PG-functionalized LAGs (PG-LAGs) in HCT116 colorectal cancer cells and normal human fibroblasts and the derived selectivity index (SI)

**Preparations**	**IC50 in HCT-116 cell line (µg/mL)**	**IC50 in normal fibroblasts (ug/mL)**	**Selectivity index (SI)**
5-FU	1.48 ± 0.19	12.77 ± 1.04	8.63
PG	2.03 ± 0.17	36.28 ± 6.91	17.87
LAGs	393.44 ± 4.20	11,110.0 ± 59.21	28.24
PG:BG (1:25)	46. 47 ± 1.74	2027.37 ± 92.15	43.60

Table 2 shows values for the Combination Index (CI) and Dose Reduction Index (DRI). CI is a parameter used to indicate synergistic (CI < 1), additive (CI = 1), or antagonistic (CI > 1) effects of 2 drugs in combination while the Dose Reduction Index (DRI) expresses the synergy of two drugs and indicate the fold-decrease in the dose of each drug independently related to their dose in the combination. Both CI and DRI values were generated by analysis of the combinatorial cytotoxic effect of PG-LAGs on HCT116 cells following a 24 h treatment at EC50 (effective dose for 50% cell viability inhibition achieved by the combination). The CI was 0.997, pointing to a potential synergistic cytotoxic effect of PG and LAGs. The concentrations of PG and LAGs as single components at EC50 were reduced by their combination in PG-LAGs, producing DRI values > 1 with 1.13 – and 8.81-fold dose reduction for PG and LAGs, respectively.

**Table 2:** Combination index (CI) and dose reduction index (DRI) generated by CompuSyn analysis of the combined cytotoxic effects of prodigiosin (PG) and *L. acidophilus* ghosts (LAGs) either singly or in combination as PG-LAGs (1:25) on HCT116 CRC cell line for 24 h at EC50 (effective dose for 50% cell viability inhibition by the combination). PG concentration range 0.25–10 μg/mL

**EC**	**Combination index (CI)**	**Concentration of PG and LAGs as single agents**		**Concentration of PG and LAGs combined** **in PG-LAGs**		**Dose Reduction Index (DRI)**	
		**PG** **(µg/mL)**	**LAGs** **(µg/mL)**	**PG** **(µg/mL)**	**LAGs (µg/mL)**	**PG**	**LAGs**
50	0.997	2.03	393.44	1.79	44.68	1.13	8.81

#### Analysis of HCT116 Cells Apoptosis Following the Incubation with LAGs

**Fig. 9:**
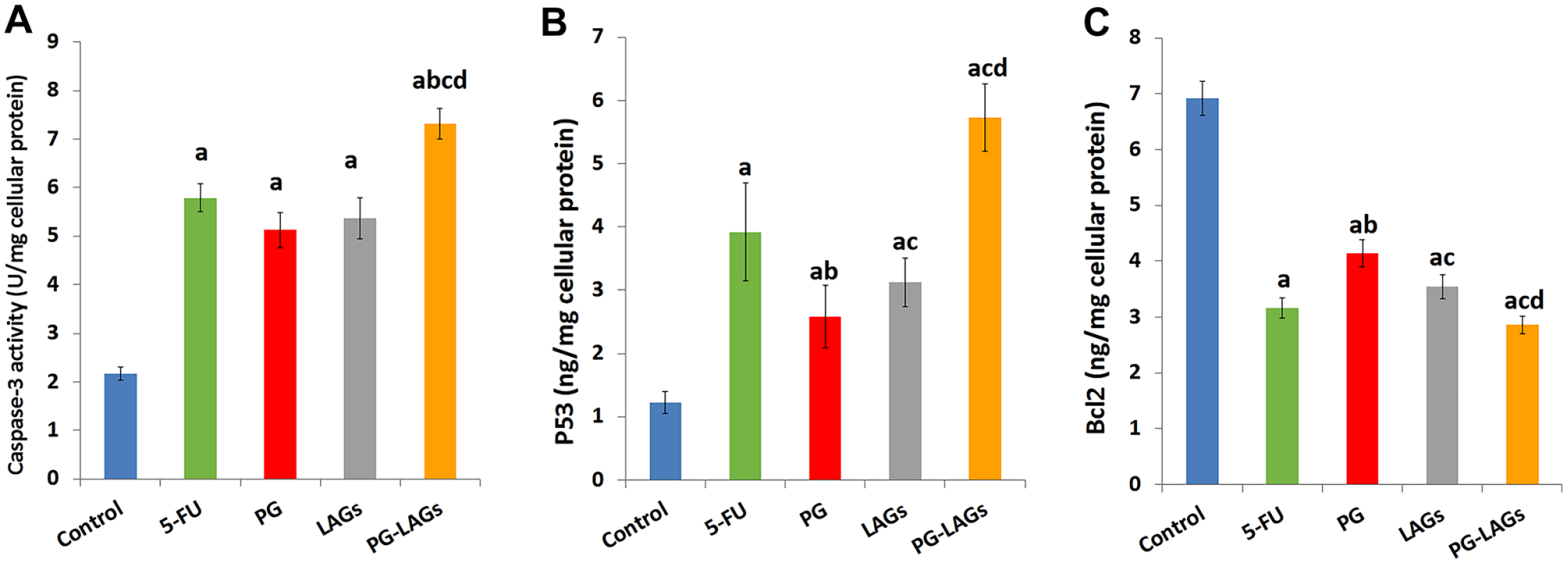
The effect of test preparations on apoptosis-related biomarkers. The effect of 5-FU (1.48 µg/mL), PG (2.03 µg/mL), LAGs (393.44 µg/mL), and PG-LAGs (46.47 µg/mL total dose) in comparison with control on the level of (**A**) Caspase 3 determined colorimetrically and **B** P53 and **C** Bcl-2, both determined by ELISA assay. Protein levels were estimated per mg of HCT116 cellular protein. Data points represent the mean ± SEM (*n* = 3) (a), (b), (c), and (d) (*p* < 0.05) indicate a significant difference vs the corresponding control, 5-FU, LAGs, and PG, respectively

The effect of PG, LAGs, and PG-LAGs in comparison with 5-FU on the level of three apoptosis-related biomarkers, namely caspase-3, P53, and Bcl-2 per mg of HCT116 cellular protein is illustrated in Fig. 9A-C. Regarding caspase 3 activity, Fig. 9A indicated a significant increase (*p* < 0.05) in the activity of the apoptotic caspase 3 by all treatments relative to control. The difference between single treatments was not statistically significant. However, PG-LAGs exerted a significantly greater (*p* < 0.05) increase in caspase 3 activity relative to its single PG and LAGs components and 5-FU. As shown in Fig. 9B, expression of the pro-apoptotic P53 protein was significantly increased (*p* < 0.05) by all treatments, although the greatest upregulating effect was exerted by PG-LAGs. The PG-LAGs effect was significantly greater (*p* < 0.05) than that of its single components but not 5-FU. On the other hand, the level of the anti-apoptotic cellular Bcl-2 protein (Fig. 9C) was significantly (*P* < 0.05) reduced by all treatments. However, reduction by PG-LAGs was significantly greater (*P* < 0.05) than that exerted by its single components but not 5-FU.

## Discussion

LAGs were prepared using the chemical SLRP method [38] with modification involving replacement of H_2_O_2_ and CaCO_3_ with NaOH and SDS at their respective MGC and MIC. This generated a bacterial mass containing LAGs in addition to un-evacuated dead LA cells and live LA cells which resisted chemical lysis. Chemical treatment was performed on bacterial cells harvested in the late stationary phase to avoid damage of the cell envelope. LAGs obtained were intact probably because of increased cross linking of the peptidoglycan layer during the late stationary phase, conferring stiffness to the cell wall [54]. Resistance of some bacterial cells to chemical lysis can be attributed to the hydrophobicity of the LA envelope [55].

Differentiation of LAGs from un-evacuated dead LA cells and live LA cells by a new method based on toluidine blue, a basic thiazine molecule known to highly bind to nucleic acids [56] indicated incomplete lysis of LA cells. Attempts to increase the proportion of LAGs in the mass by increasing NaOH and SDS concentrations or preparing LAGs under static conditions resulted in cell envelope rupture (Fig. 1e, f). Accordingly, the product of chemical lysis was a bacterial mass consisting of LAGs in combination with live and dead un-evacuated LA cells. A purified fraction of LAGs was successfully separated from the bacterial mass by density gradient centrifugation [42], a method not documented to date for the separation of ghosts from un-evacuated bacterial cells. Verified purity of the separated ghost fraction (Fig. 2) ascertained the efficiency of density gradient centrifugation as a practical method for the separation of ghosts from a bacterial mass.

Characterization of the purified LAGs by SEM and TEM, gel electrophoresis, and light microscopy (Fig. 2 and supplementary Fig. 1) collectively verified the morphology of LAGs as empty vesicles devoid of cytoplasmic content with an internal volume of 0.13 µm^3^ approximately and surrounded by an intact and cohesive negatively charged cell wall. Pores with a mean size of 153.63 ± 12.23 nm were located at the division sites representing the weak point of the wall. Moreover, SDS-PAGE bands at 46 KDa and 49 KDa indicated the existence of the S-layer in LAGs similar to live LA cells, implying preservation of a surface structural component of importance to cellular adhesion [57, 58]. This difference in molecular weight can be attributed to the difference in growth phases during which cells were harvested [59]. Proteins of lane 1 and lane 2 (supplementary Fig. 1b) were obtained from cells harvested during the exponential phase and the late stationary phase respectively before chemical treatment. The lighter band intensity at 49 KDa in lane 2 could be explained by partial detachment of some surface proteins as a result of chemical treatment.

The demonstrated LAGs properties would have important implications in their bioactivity. For instance, retention of the intact capsule around LAGs is a crucial factor in adhesion to cellular membranes, a process mediated by the capsular polysaccharides cohesive layer [60]. Moreover, potential retention of teichoic and lipoteichoic acids as indicated by surface negativity of LAGs would contribute to the integrity of the cell membrane [61] and adhesion to colon cells [62, 63]. Agarose gel electrophoresis and quantitative data obtained by Nanodrop^®^ spectrophotometry indicated the elimination of most genetic materials from LAGs.

Applicability of LAGs in biotargeted drug delivery was supported by a relatively large intracellular space of 0.13 µm^3^ and a negatively charged intact membrane as potential sites for drug loading in addition to surface characteristics favoring inherent tropism for colon cells. In addition, LAGs exhibited a relatively small size (1.13 ± 0.13 µm) and size uniformity (PDI 0.15 ± 0.09). The potential of LAGs as bioinspired anti-CRC delivery system was assessed using PG, a secondary bacterial metabolite with established apoptotic activity against CRC. However, the development of PG-functionalized LAGs was challenged by the high hydrophobicity of PG (log *P*_octanol-water_ 5.16) and its poor solubility in aqueous physiological media, a well-documented PG formulation problem [64–66]. PG loading into LAGs by a simple incubation method and agitation at 200 rpm was influenced by the proportion of acetic acid in the methanol-acetic acid solvent system used, the incubation time, and PG concentration. Loading of PG into LAGs was enhanced by increasing the PG concentration in the input solution (supplementary Fig. 2) and was verified by digital photography and light microscopy (Fig. 5e, f). Failure of eight rounds of washing with 0.5% NaCl and centrifugation to remove PG from LAGs surface implied strong affinity of PG to LAGs. PG loading efficiency was 4% (payload 40 µg/mg of LAGs).

The composition of the solvent system appears to play a key role in PG loading into LAGs and maintaining its entrapment stability. It has been reported that short chain alcohols modulate the properties of phospholipid bilayers promoting their permeabilization [67, 68]. By promoting PG protonation [69, 70], the acetic acid component potentially enhances PG binding to the negatively charged LAG membrane via strong electrostatic interaction with head groups of teichoic and lipoteichoiuc acids. Localization of PG in the LAGs cell envelopes was verified by TEM (Fig. 5g, h) and CFM imaging (Fig. 6).

The lack of in vitro release of PG in buffers with different pH and composition and simulated gastrointestinal fluids at 100 rpm and 37 °C provided further evidence for the retention of PG by LAGs. High stability of the PG-LAGs structure in physiological media and negligible PG release was confirmed by the recovery of 93.15 ± 8.8% of loaded PG from LAGs at the end of a 48 h release experiment using PBS pH 7.4 containing 3% Tween 80. The structural stability of PG-LAGs and potential localization of PG molecules within the resistant ghost capsule and electrostatic interaction with the negatively charged cell wall components appear to have precluded PG release. PG and other hydrophobic bioactive agents were reported to interact with lipophilic components of the cell membranes of bacterial cells and ghosts [64, 71]. Drug release from BGs was shown to depend mainly on the type of ghost and the drug physicochemical properties. While *E. coli* ghosts failed to release resveratrol [71], 12% of doxorubicin was released from *Mannheimia haemolytica* ghosts in 10 h [51]. Drug release properties of bacterial ghosts (BGs) may differ greatly from those of polymer-based drug carriers which usually undergo marked physicochemical changes under the release conditions contributing to drug liberation. For instance, sustained release of PG from PLGA (poly D,L-lactic-co-glycolic acid) microparticles in PBS pH 7.4 containing 0.1% w/v Tween 80 at 37 °C was mediated by a combination of diffusion-, dissolution- and polymer degradation-controlled mechanisms [66]. Similarly, release of PG from chitosan microspheres at pH 7.4 involved porosity and degradation of the polymer matrix [72]. Release data in the present study implied strong PG binding to LAGs, probably constituting a single bioactive entity.

Despite the lack of PG release from LAGs, PG-LAGs were active at both the cellular and molecular levels against HCT116, a human-derived cell line reflecting the major molecular characteristics of colorectal cancer. Cytotoxicity data provided a proof of concept for potential application of PG-LAGs as an anti-CRC bacterial structure. It is worth noting that trafficking and targeting of drugs by bacterial structures may involve mechanisms not necessarily dependent on extracellular drug delivery [11]. Mechanisms include fusion of the bacterial structure with cell membranes of the target cancer cells with subsequent release of their cargo inside the cytoplasmic space [73, 74]. Uptake of bacterial ghosts by other types of cells such as antigen presenting cells [75] and conjunctival epithelial cells [76] has been reported.

Indeed, cell viability data obtained following 24 h treatment of HCT116 cells (Fig. 8a-d), a model for CRC initiating cells with stem-like cells properties [34] generated multiple important findings. These comprise a PG cytotoxic effect approaching that of 5-FU, the first-line treatment for colorectal cancer (CRC), with insignificantly different IC50 values. Such a considerable activity is explained by the well-established effect of PG against CRC cells [65] in addition to its apoptotic activity against cancer stem cells [34, 36] which account for a relatively large proportion of HCT116 cells [77]. In contrast, 5-FU does not inhibit CRC stem cells [78]. Interestingly, native LAGs exerted a cytotoxic effect against HCT116 cells, corroborating data *for E. coli* ghosts against Caco-2 cell line [24]. This is a valuable merit of LAGs as bioactive carrier for colon targeting as some bacterial ghosts might exert a cancer cell proliferating effect [51]. The cytotoxic activity of LAGs was significantly enhanced by PG loading, achieving ≈ 8.5-fold reduction in LAGs IC50 as well as an increase in their selectivity for HCT116 cancer cells relative to NHFs used as model noncancerous cells [79, 80], surpassing that of 5-FU (Table 1). The notable safety of LAGs to NHF cells, a commonly used model for assessing drug safety, their inherent tropism for colon cells and possible cellular uptake may account for the relatively high selectivity of PG-LAGs for CRC cells. Importantly, analysis of the combinatorial anticancer effects of PG and LAGs in PG-LAGs following a relatively short 24 h incubation with HCT116 cells pointed to potential synergism that was associated with an anticipated 1.13-fold and 8.81-fold reduction in the dose of PG and LAGs respectively in the combination at EC50 (Table 2).

The anticancer merits of PG-LAGs demonstrated at the cellular level were substantiated at the molecular level by significant modulation of the levels of three apoptosis-related biomarkers (Fig. 9A-C). PG and LAGs in comparison with 5-FU showed a significant increase in the intracellular apoptotic caspase 3 activity and P53 protein levels and significant downregulation of the anti-apoptosis-related B-cell lymphoma 2 ( Bcl-2) protein level relative to untreated control cells. Upregulation of caspase 3 activity and apoptosis of HCT116 and HT-29 CRC cells [34, 35] by PG appear to depend on a decrease in the mRNA and protein levels of the proto-oncogene survivin, providing a potential molecular mechanism for PG-induced apoptosis. P53, a tumor suppressor frequently mutated or inactivated in colorectal cancer, was significantly more upregulated by PG-LAGs compared to PG, LAGs, and 5-FU (Fig. 9b). A possible mechanism might involve enhancement of the PG-induced restoration of the p53 pathway known to target CRC stem cells which represent a considerable proportion of HCT116 cells via activation of p73, a member of the p53 family [36], leading to cell growth inhibition. Bcl-2 is known to exert an ant-apoptotic effect, supporting drug resistance in cancer cells [81]. Significant suppression of Bcl-2 level in HCT116 cells by PG-LAGs contributed to their apoptotic effect. Activation of caspase 3 combined with upregulation of P53 and downregulation of Bcl-2 demonstrated a high pro-apoptotic capacity of PG-LAGs in the treated HCT116 cells. LAGs-induced molecular effects supported the intrinsic tropism and cytotoxicity of LAGs against CRC cells, an issue warranting further investigation.

The significantly greater modulating effect of PG-LAGs relative to PG and LAGs can be explained by enhanced intracellular activities as a result of fusion or uptake of PG-LAGs by the HCT116 CRC cells. Results highlighted activity of LAGs against CRC cells, promoting their utilization as a novel bacterial ghost carrier for colon targeting in addition to the potentials of PG-LAGs in the treatment of colorectal carcinoma.

## Conclusion

The current study provides new methodological information for the generation of highly purified ghosts of the Gram-positive LA using a density gradient centrifugation technique not documented to date and the differentiation of ghosts from un-evacuated bacterial cells using a simple staining method. Functionalization of the produced LAGs with PG generated a novel microbially derived bioactive structure with high membrane integrity and stability under physiological conditions. Significant activity against CRC HCT116 cells at the cellular and molecular levels provided a proof of concept for the application of PG-LAGs as an anti-CRC delivery system combining the safety, bioactivity, and inherent affinity of probiotic LAGs for colon cells and the apoptotic activity of PG.

## Supplementary Information

Below is the link to the electronic supplementary material.

Supplementary file1 (DOCX 1234 KB)
