# Supplementary material for: Prodigiosin-Functionalized Probiotic Ghosts as a Bioinspired Combination Against Colorectal Cancer Cells
Source: Probiotics Antimicrob Proteins. 2022 Aug 28;15(5):1271–86. doi: 10.1007/s12602-022-09980-y (PMC10491537; doi:10.1007/s12602-022-09980-y)
Supplement: Supplementary file 1 — Supplementary file1 (DOCX 1234 KB) [file 12602_2022_9980_MOESM1_ESM.docx]

**Prodigiosin-functionalized the probiotic *Lactobacillus acidophilus* ghosts as a bioinspired combination against colorectal cancer cells**

**Probiotics and Antimicrobial Proteins**

Nessrin Saleh^1^, Hoda E. Mahmoud^1^, Hoda Eltaher^2 a, b^, Maged Helmy^3^, Labiba El-Khordagui^2 a*^, Ahmed A. Hussein^1^

^1^ Department of Biotechnology, Institute of Graduate Studies and Research, Alexandria University, Alexandria, Egypt. ^2a^ Department of Pharmaceutics, Faculty of Pharmacy, Alexandria University, Alexandria, Egypt, 21521. ^2b^ Regenerative Medicine and Cellular Therapies Division, Faculty of Science, University of Nottingham, University Park, Nottingham NG7 2RD, UK. ^3^ Department of Pharmacology and Toxicology, Faculty of Pharmacy, Damanhour University, Damanhour, Egypt

***Corresponding author:**

Labiba El-Khordagui, E-mail: [labiba.elkhordagui@alexu.edu.eg](mailto:labiba.elkhordagui@alexu.edu.eg) Tel: +201005550567

ORCID: 0000-0002-6607-8113

**Supplementary material**

**a**


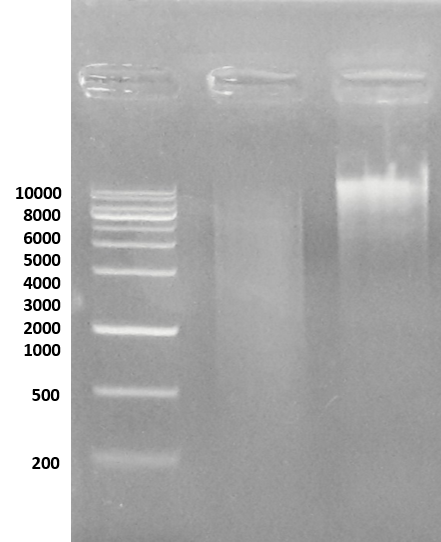


**M**

**1**

**2**


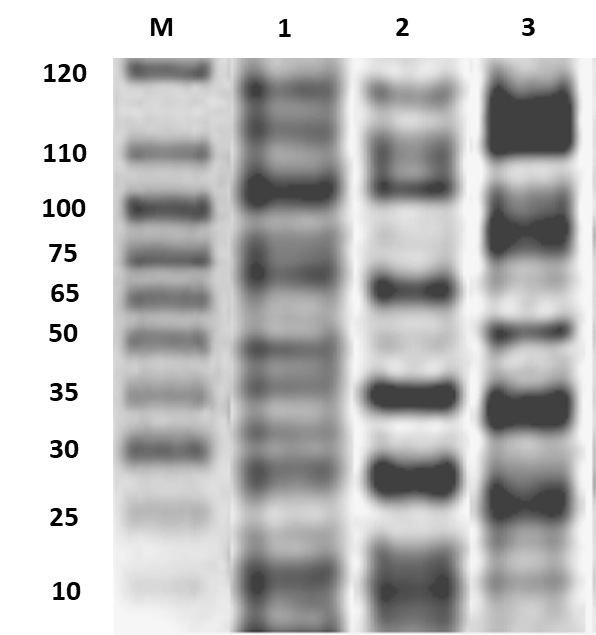


**b**

**AA**

**Supplementary Figure 1**. Detection of DNA and proteins in live *L. acidophilus* (LA) and their ghosts (LAGs). a: DNA profile on agarose gel under UV transilluminator showing: M. 1 kbp marker, lane 1: DNA isolated from purified LAGs and lane 2: DNA isolated from live LA cells before chemical treatment; b: SDS-PAGE stained with Coomassie brilliant blue R250 for proteins of live LA cells, LAGs, and the supernatant after chemical treatment. M: Molecular weight marker, lane 1: LA cell proteins, Lane 2: LAG proteins and Lane 3: Intracellular proteins in the supernatant after chemical treatment.


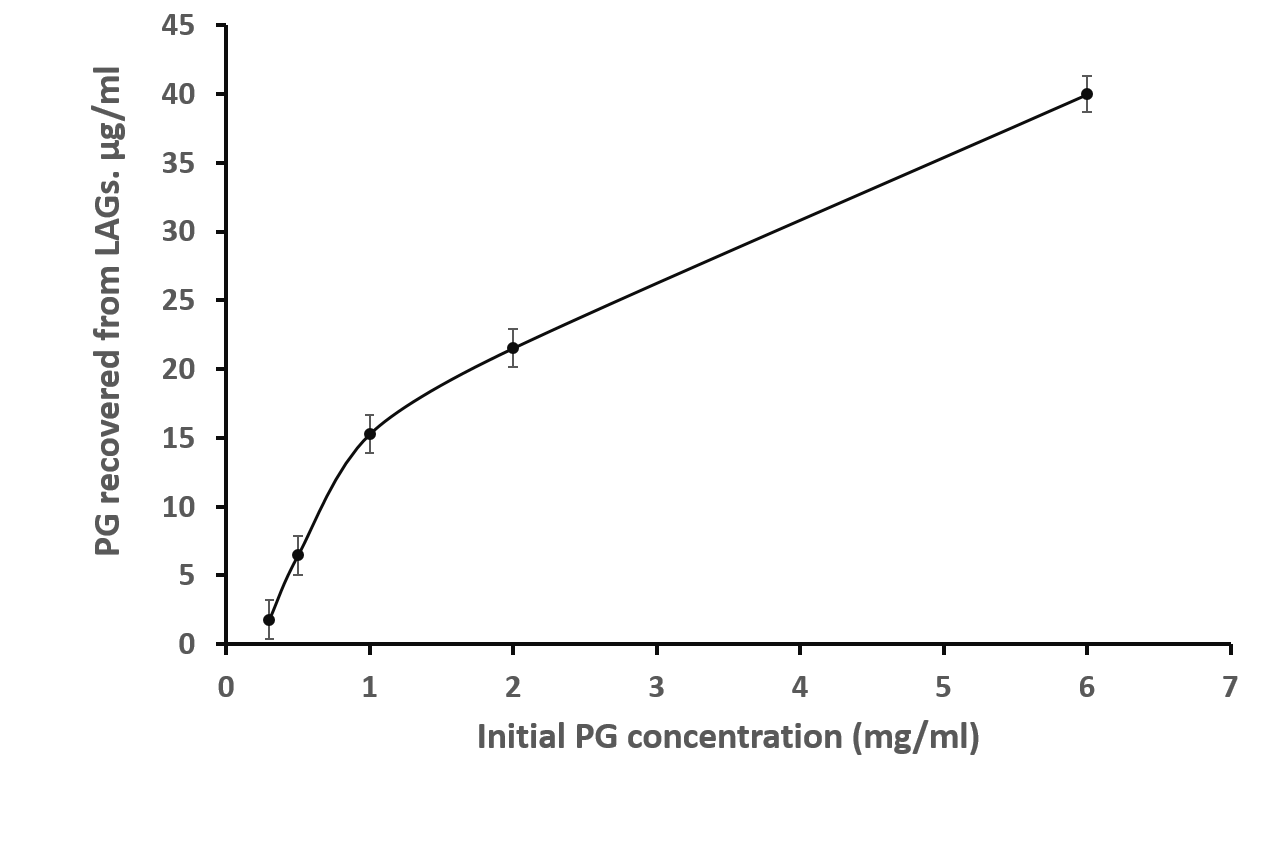


**Supplementary Figure 2** Effect of concentration of the input PG solution in methanol-acetic acid (1:1) solvent blend on the concentration of PG loaded into LAGs following 2h-incubation with agitation at 200 rpm at ambient temperature, recovered by vigorous shaking with methanol. Error bars represent SD (n=3).

Supplementary Table 1. Data matrix for protein fingerprinting patterns based on band % for protein marker, lane 1: LA cell proteins, Lane 2: LAG proteins and Lane 3: Intracellular proteins in the supernatant after chemical treatment.

| Protein marker | | 1 | | 2 | | 3 | |
| --- | --- | --- | --- | --- | --- | --- | --- |
| Lane % | MW | Lane % | MW | Lane % | MW | Lane % | MW |
| 7.88 | 120.000 | 8.56 | 117.598 | 6.71 | 116.918 | 13.11 | 114.584 |
| 10.52 | 110.000 | 8.72 | 112.667 | 8.94 | 110.000 | 11.8 | 111.746 |
| 13.74 | 100.000 | 9.64 | 106.124 | 9.29 | 105.578 | 2.88 | 104.321 |
| 10.66 | 75.000 | 4.22 | 101.954 | 7.86 | 67.109 | 2.68 | 100.000 |
| 10.24 | 65.000 | 4.98 | 73.158 | 2.71 | 49.010 | 6.31 | 89.555 |
| 9.39 | 50.000 | 4.15 | 67.744 | 13.52 | 35.000 | 3.22 | 69.597 |
| 9.72 | 35.000 | 7.59 | 46.211 | 12.44 | 28.226 | 7.27 | 53.116 |
| 13.30 | 30.000 | 7.42 | 36.949 | 4.98 | 27.394 | 9.90 | 33.447 |
| 7.45 | 25.000 | 5.52 | 31.062 | 2.89 | 12.695 | 6.64 | 32.505 |
| 4.41 | 10.000 | 10.49 | 28.753 | 13.55 | 8.846 | 7.29 | 27.619 |
|  |  | 3.04 | 20.774 |  |  | 13.60 | 26.037 |
|  |  | 8.22 | 11.351 |  |  | 6.12 | 10.676 |
|  |  | 4.88 | 8.846 |  |  |  |  |
